# Intranasal influenza vaccination using a new synthetic mucosal adjuvant SF‐10: induction of potent local and systemic immunity with balanced Th1 and Th2 responses

**DOI:** 10.1111/irv.12124

**Published:** 2013-05-26

**Authors:** Takashi Kimoto, Dai Mizuno, Tsunetomo Takei, Takuya Kunimi, Shinji Ono, Satoko Sakai, Hiroshi Kido

**Affiliations:** ^1^ Division of Enzyme ChemistryInstitute for Enzyme ResearchThe University of TokushimaTokushimaJapan

**Keywords:** influenza vaccine, nasal vaccination, Pulmonary surfactant, synthetic mucosal adjuvant, Th1/Th2 responses

## Abstract

**Background:**

We found previously that bovine pulmonary Surfacten^®^ used in newborns with acute respiratory distress syndrome is a safe and efficacious antigen vehicle for intranasal vaccination.

**Objectives:**

The objective of this study was to industrially produce a synthetic adjuvant mimicking Surfacten^®^ for clinical use without risk of bovine spongiform encephalopathy.

**Methods:**

We selected three Surfacten lipids and surfactant protein (SP)‐C as essential constituents for adjuvanticity. For replacement of the hydrophobic SP‐C, we synthesized SP‐related peptides and analyzed their adjuvanticity. We evaluated lyophilization to replace sonication for the binding of influenza virus hemagglutinin (HA) to the synthetic adjuvant. We also added a carboxy vinyl polymer (CVP) to the synthetic adjuvant and named the mixture as SF‐10 adjuvant. HA combined with SF‐10 was administered intranasally to mice, and induction of nasal‐wash HA‐specific secretory IgA (s‐IgA) and serum IgG with Th1‐/Th2‐type cytokine responses in nasal cavity and virus challenge test were assessed.

**Results and Conclusions:**

Intranasal immunization with HA–SF–10 induced significantly higher levels of anti‐HA‐specific nasal‐wash s‐IgA and serum IgG than those induced by HA‐poly(I:C), a reported potent mucosal vaccine, and provided highly efficient protection against lethal doses of virus challenge in mice. Anti‐HA‐specific serum IgG levels induced by HA–SF–10 were almost equivalent to those induced by subcutaneous immunization of HA twice. Intranasal administration of HA–SF–10 induced balanced anti‐HA‐specific IgG1 and IgG2a in sera and IFN‐γ‐ and IL‐4‐producing lymphocytes in nasal cavity without any induction of anti‐HA IgE. The results suggest that HA–SF–10 is a promising nasal influenza vaccine and that SF‐10 can be supplied in large quantities commercially.

## Introduction

The recent global spread of swine‐origin H1N1 influenza A virus (IAV) highlighted the need for the development of effective vaccines for the prevention of viral infection and transmission. The currently available influenza vaccines administered intramuscularly or subcutaneously induce a predominantly IgG‐mediated protection in the systemic immune compartment and significantly reduce hospitalization and deaths when they match antigenically the circulating viral strains.^1^, ^2^ However, this immunization results in neither adequate induction of antiviral secretory IgA (s‐IgA), which provides a wide cross‐protection, nor efficient prevention of the initial airway infection^3^, ^4^ or cell‐mediated responses in the upper and lower respiratory tracts.^5^ To address the need for improved influenza vaccines, nasal vaccination that stimulates both mucosal and systemic immunity^4^ is desirable for future vaccination against airway viral infection.

Mucosal vaccines and adjuvants have been studied for over 40 years^6^, ^7^; however, many were found to be ineffective or have safety problems.^8^ There are two key issues with regard to nasal vaccines.^9^ One is the poor efficiency of antigen uptake across the nasal mucosa due to mucociliary clearance. The other is the safety issue; that is, protection against hyperstimulation of antigen‐presenting cells or unexpected antibody induction. At present, the cold‐adapted live flu mucosal vaccine, FluMist^®^, is available in the USA^10^ for individuals aged 2–49 years and has been licensed in Europe from 2012 under the name FLUENZ for children aged 2–17 years. It is not licensed for children <2 years of age where it is known to potentially cause post‐vaccination flu symptoms with severe wheezing.^11^, ^12^

To overcome these issues, we recently reported that natural pulmonary surfactant and its commercially available bovine product, Surfacten^®^, which has been used as a natural replacement medicine in premature babies for more than 25 years without significant adverse effects,^13^ show safety and efficacy of mucosal adjuvanticities by promoting antigen delivery to antigen‐presenting cells in mice and minipigs.^9^, ^14^, ^15^ The lung surfactant is effectively uptaked into alveolar cells, macrophages, and dendritic cells and rapidly metabolized *in vivo* with a short half‐life of 6–7 hour in the lungs.^16^ In addition, we recently found that three major Surfacten lipids and surfactant protein C (SP‐C) are essential constituents for mucosal adjuvanticity of Surfacten.^9^ In mammals, SP‐C is a 33‐ to 35‐residue lipopeptide that consists of a hydrophobic transmembrane α‐helix and a cationic N‐terminal segment and plays an important role in the uptake of surfactant lipids to alveolar macrophages and epithelial cells.^17^ To provide ample supply of the mucosal adjuvant for clinical use, it is important to commercially develop a synthetic compound that carries no risk of bovine spongiform encephalopathy.

In this study, we describe an effective preparation process of a synthetic surfactant (SSF) and a further improved synthetic mucosal adjuvant SF‐10 mimicking Surfacten for large‐scale manufacturing by improving the following issues. As a substitute for SP‐C(1–35), which does not dissolve easily in common organic solvents, we identified a methanol‐soluble SP‐related peptide. The formation of a complex between influenza hemagglutinin vaccine (HA) and SSF was improved for large‐scale manufacturing by lyophilization instead of sonication. In addition, we found a mucoadhesive additive to increase the viscosity of HA‐SSF mixture to avoid rapid clearance from the nasal cavity and termed the improved adjuvant compound SF‐10. Based on these improvements, we analyzed the enhancement of mucosal and systemic immunity by SF‐10 and the resultant Th1‐ and Th2‐type cytokine responses and protective immunity in mice.

## Materials and methods

### Animals and virus

All experiments were performed in 6‐ to 8‐week‐old BALB/c female mice obtained from Japan SLC, Inc. (Shizuoka, Japan). All animals were treated according to the Guide for the Care and Use of Laboratory Animals (NIH Publication No. 85‐23, 1996), and the study was approved by the Animal Care Committee of the Tokushima University. IAV/ PR8/34(H1N1) and A/New Caledonia/20/99(H1N1) were provided by The Research Foundation for Microbial Diseases of Osaka University (Kagawa, Japan).

### Reagents

Surfacten^®^ was purchased from Mitsubishi Pharma (Tokyo, Japan). 1,2‐Dipalmitoyl‐phosphatidylcholine (DPPC), phosphatidylglycerol (PG), and palmitate (PA) for the preparation of SSF were obtained from Nippon Fine Chemical (Osaka, Japan). Synthetic SP‐related peptides (Table 1; >80% grade) were obtained from Greiner (Frickenhausen, Germany). A carboxy vinyl polymer (CVP) was purchased from Sigma‐Aldrich (St. Louis, MO, USA) and poly(I:C) from Alexis Biochemicals (Lausen, Switzerland).

**Table 1:** Amino acid sequences of peptides derived from SP‐B and SP‐C

	Amino acid sequence
SP‐B‐type peptide	
SP‐B(1–25)	FPIPLPYCWLCRALIKRIQAMIPKG
SP‐B(20–60)	AMIPKGALAVAVAQVCRVVPLVAGGICQCLAERYSVILLDT
SP‐B(64–80)	RMLPQLVCRLVLRCSMD
KL4	KLLLLKLLLLKLLLLKLLLLK
SP‐C‐type peptide	
SP‐C(1–35)	FGIPCCPVHLKRLLIVVVVVVLIVVVIVGALLMGL
SP‐C(1–12)	FGIPCCPVHLKR
SP‐C(1–19)	FGIPCCPVHLKRLLIVVVV
SP‐C(13–35)	LLIVVVVVVLIVVVIVGALLMGL
SP‐CL11	PVHLKRLLLLLLLLLLL
SP‐CL16	PVHLKRLLLLLLLLLLLLLLLL
K6L16	KKKKKKLLLLLLLLLLLLLLLL

### Preparation of antigen and SSF

IAV/New Caledonia/20/99(H1N1) split antigen (0·2 μg of viral protein, corresponding to 0·14 μg HA; Denka Seiken, Tokyo, Japan) was used as a HA antigen in the present studies. SSF was prepared by mixing three lipids, DPPC, PG, and PA, and various SP‐related synthetic peptides, at a molar ratio of 75:25:30:0·6, respectively, as described previously.^9^ SSF samples (4 mg phospholipids/ml) were then lyophilized for storage.

### Immunization and virus challenge

Lyophilized SSF (its weight was expressed as that of phospholipids) was suspended in distilled water and then mixed with HA (its weight was expressed as protein) in saline at a ratio of 10:1 (wt/wt) as described previously.^9^ The SSF–HA complex formation was carried out by sonication or lyophilization. Sonication method^9^: A mixture of SSF and HA was treated for 3 minute in a sonic oscillator (model S‐250D; Branson Ultrasonics, Danbury, CT, USA) followed by upside‐down mixing every 30 minute for 2 hour at room temperature and then stored at 4°C.

#### Lyophilization method

A mixture of SSF and HA was incubated at 42°C, the critical temperature of Surfacten lipids, for 10 minute with gentle mixing, followed by freezing at −75°C, and then lyophilized. Lyophilized HA + SSF was dissolved in saline before use. Just before administration to mice, CVP in saline at pH 7·0 was added to HA‐SSF solution at a final concentration of 0·5%, and the final solution (HA + SSF + CVP) was renamed as HA–SF–10. The amount of HA bound to SSF was calculated by estimating the amount of unbound HA in the SSF‐free supernatant fraction after centrifugation as described previously.^9^ However, the percentage binding of HA to SF‐10 could not be measured due to the inability to separate unbound HA from HA–SF–10 complex by centrifugation or ultrafiltration because of increased viscosity after the addition of CVP.

Mice were immunized by intranasal instillation two or three times every 2 weeks with the prepared samples (2 μl) into each nostril. Positive control mice were subcutaneously injected HA in 100 μl saline under the above immunization schedule. Two weeks after the last immunization, serum and nasal washes were prepared as described previously.^9^, ^14^

For virus challenge experiments, immunized mice were infected with IAV/New Caledonia/20/99(H1N1) at 5 × 10^4^ plaque‐forming unit (PFU) by intranasal instillation, for the measurement of neutralization activities of nasal washes. At day 4 after the challenge, virus titers were measured in nasal and lung washes by the plaque assay using Madin‐Darby canine kidney cells, which is based on the detection of infected cells using anti‐IAV nucleoprotein monoclonal antibody, as described previously.^18^

Immunized mice were also infected with lethal doses of highly pathogenic IAV/ PR8/34(H1N1; 50 and 800 PFU) in 20 μl saline, and the survival rate was monitored for 14 days.

### Analysis of mucosal immune responses by ELISA and ELISPOT

Two weeks after the last immunization, serum and nasal‐wash specimens were prepared as described previously^14^ and subjected to enzyme‐linked immunosorbent assay (ELISA) to determine anti‐HA‐specific s‐IgA, IgG, IgG1, IgG2a, and IgE levels.^9^, ^14^, ^15^ We used goat anti‐mouse IgA, IgG (Sigma, St. Louis, MO, USA), IgG1, IgG2a, or IgE (Bethyl Laboratories, Montgomery, TX, USA) antibodies conjugated with horseradish peroxidase as the secondary antibodies. The levels of total IgE were determined by mouse IgE ELISA Quantitation Set (Bethyl Laboratories). We used 50 ng/ml of purified mouse anti‐HA s‐IgA, IgG, IgG1, and IgG2a antibodies, which were prepared as described previously,^14^ as standards. The hemagglutination inhibition (HI) titers in serum were analyzed after treatment of samples with receptor‐destroying enzyme, and the assay was conducted according to the protocol for HI testing established by the World Health Organization, as reported previously.^15^

Mononuclear cells isolated from nasal passages and nasopharynx‐associated lymphoid tissue (NALT) by discontinuous Percoll (GE Healthcare, Buckinghamshire, England) density gradient centrifugation^19^ were subjected to enzyme‐linked immunosorbent spot (ELISPOT) assay, as described previously.^18^ The numbers of IL‐4‐ and IFN‐γ‐producing cells were counted by Mouse ELISPOT Set for IL‐4 (BD Pharmingen, Franklin Lakes, NJ, USA) and IFN‐γ (MABTECH, Nacka Strand, Sweden) according to the protocols provided by the manufacturers.

### Neutralizing activity

To assess the neutralizing activities of the s‐IgA in nasal washes, the s‐IgA fraction was purified with KAPTIV‐AE™ IgA affinity column according to the protocol provided by the manufacturer (Tecnogen, Piacenza, Italy). The concentration of s‐IgA was measured by Mouse IgA ELISA Quantitation Set (Bethyl Laboratories). IAV/New Caledonia/20/99(H1N1) at 200 PFU was incubated with 100 μl of the serially diluted s‐IgA at 37°C for 1 hour and then the virus titers in the mixtures were measured by the plaque assay, as described previously.^9^

### Statistical analysis

All data were expressed as mean ± SD. Differences between groups were examined for statistical significance using the unpaired Student's *t*‐test. A *P* value less than 0·05 denoted statistical significance.

## Results

### Improvement of synthetic mucosal adjuvant SSF for ample supply

We reported previously that SP‐C but not SP‐B is an essential constituent of Surfacten for mucosal adjuvanticity.^9^ SP‐C(1–35) with hydrophobic properties, however, is soluble in 100% trifluoroacetic acid but not in common organic solvents, and its industrial production is scarce. Thus, we designed various peptide fragments of SP‐C and SP‐B and their modifications (Table 1). SSFs were synthesized by mixing three lipid constituents and each synthetic peptide, and the adjuvanticity was analyzed (Table 2). Adjuvanticity of SSFs containing SP‐C‐related peptides with 11 to 16 hydrophobic amino acid chain length, but not 7, was almost equivalent to that of Surfacten. Although SSF containing the C‐terminal‐side hydrophobic domain peptide SP‐C(13–35) showed relatively high values of anti‐HA‐specific s‐IgA in nasal washes and anti‐HA‐specific IgG in serum, the levels were not consistent with large SD values. SP‐C‐related peptides with 11 to 16 hydrophobic amino acid chain length and basic residues in the N‐terminal side were soluble in methanol or ethanol, and their adjuvanticity was equivalent to that of Surfacten. Based on these results, we selected a peptide K6L16 among the active peptides, SP‐C(1–35), SP‐C(13–35), SP‐CL11, SP‐CL16, and K6L16, as a substitute for SP‐C(1–35), which is soluble in methanol and easy for handling, in the following experiments.

**Table 2:** Effects of mucosal adjuvants, Surfacten, and SSF on the induction of HA‐specific antibodies

	Anti‐HA antibodies (ng/ml)	
	Nasal washes (s‐IgA)	Serum (IgG)
Saline	10·1 ± 4·5	195·5 ± 43·1
HA	9·6 ± 7·4	302·0 ± 125·9
HA‐St	196·2 ± 67·7	2907·3 ± 1465·0
HA‐SSF		
SP‐B type		
SP‐B(1–25)	52·0 ± 38·7	622·0 ± 163·9
SP‐B(20–60)	37·4 ± 29·4	853·9 ± 344·3
SP‐B(64–80)	71·1 ± 37·6	625·9 ± 121·6
KL4	88·3 ± 49·3	778·6 ± 325·9
SP‐C type		
SP‐C(1–35)	238·1 ± 122·0^a^	2628·9 ± 942·0^a^
SP‐C(1–12)	23·7 ± 25·5	798·0 ± 688·2
SP‐C(1–19)	46·4 ± 40·1	735·1 ± 398·6
SP‐C(13–35)	318·1 ± 326·6^a^	2332·0 ± 1079·7^a^
SP‐CL11	136·6 ± 85·1^a^	3851·3 ± 2164·1^a^
SP‐CL16	209·8 ± 114·3^a^	2455·2 ± 1674·3^a^
K6L16	222·7 ± 145·3^a^	2104·5 ± 941·7^a^

Mice were treated with intranasal inoculation of 0·2 μg HA with or without 2 μg of St and various SSF twice at 2‐week interval. Two weeks after the last immunization, anti‐HA‐specific s‐IgA in nasal washes and anti‐HA‐specific IgG in sera were assayed. Data are mean ± SD of 10–15 mice.

a The values are almost equivalent to those of HA‐St.

To achieve effective antigen delivery, we mixed HA and SSF by sonication to make HA‐SSF binding complex. However, avoiding heat damage to HA during sonication is difficult. Accordingly, we used lyophilization as an alternative to sonication and to remove water molecules in the interaction space between HA and SSF for the binding. Lyophilization increased the binding of HA to SSF to ≥92% compared with 65–70% by sonication and significantly increased the induced levels of anti‐HA‐specific s‐IgA in nasal washes at 11·5‐fold and IgG in sera at 168·5‐fold (*P *< 0·01), compared with those by SSF prepared by sonication (Table 3).

**Table 3:** Evaluation of various mixing procedures of HA and SSF on induction of HA‐specific antibodies

Materials			Preparation		HA binding (%)	Anti‐HA antibodies (μg/ml)	
HA	SSF	CVP	Sonication	Lyophilization		Nasal washes (s‐IgA)	Serum (IgG)
−	−	−	−	−	‐	0·0 ± 0·0	0·1 ± 0·1
+	−	−	+	−	‐	0·0 ± 0·0	0·5 ± 0·5
+	+	−	+	−	65‐70	0·2 ± 0·2	4·3 ± 0·9
+	+	−	−	+	92‐98	2·3 ± 1·4^b^	724·5 ± 248·1^b^
+	−	+	−	−	‐	0·3 ± 0·2	4·9 ± 1·6
+	+	+	+	−	‐	3·3 ± 0·9^b^	568·7 ± 95·2^b^
+	+	+	−	+	‐	9·9 ± 5·1^a^^**,**^ b	925·0 ± 633·3^b^

Mice were immunized intranasally with HA, with SSF and/or CVP in each combination, three times at 2‐week interval. Two weeks after the last immunization, anti‐HA‐specific s‐IgA in nasal washes and IgG in sera were measured by ELISA. Data are mean ± SD of 10–15 mice.

a *P* < 0·01, compared with HA‐SSF mixture prepared by sonication.

b *P *< 0·05, compared with HA‐SSF mixture prepared by lyophilization.

To increase the retention time of HA‐SSF in the nasal cavity, we added the mucoadhesive polymer CVP to HA‐SSF, to increase its viscosity, at a final concentration of 0·5% just before immunization. The addition of CVP to the lyophilization‐prepared HA‐SSF further enhanced the induction of anti‐HA‐specific s‐IgA in nasal washes significantly at 4·3‐fold (*P *< 0·05) and anti‐HA‐specific serum IgG at 1·3‐fold, the induced levels being the highest among the materials tested. Only limited inductions of anti‐HA‐specific s‐IgA and anti‐HA‐specific IgG were detected in animals immunized with HA+CPV without SSF, suggesting that SSF is an essential constituent for mucosal adjuvanticity in the mixture. The mixture of HA‐SSF and CVP, the best antigen–adjuvant complex, was renamed as HA–SF–10 in the following experiments.

### Mucosal adjuvanticity of HA–SF–10

**Figure 1:**
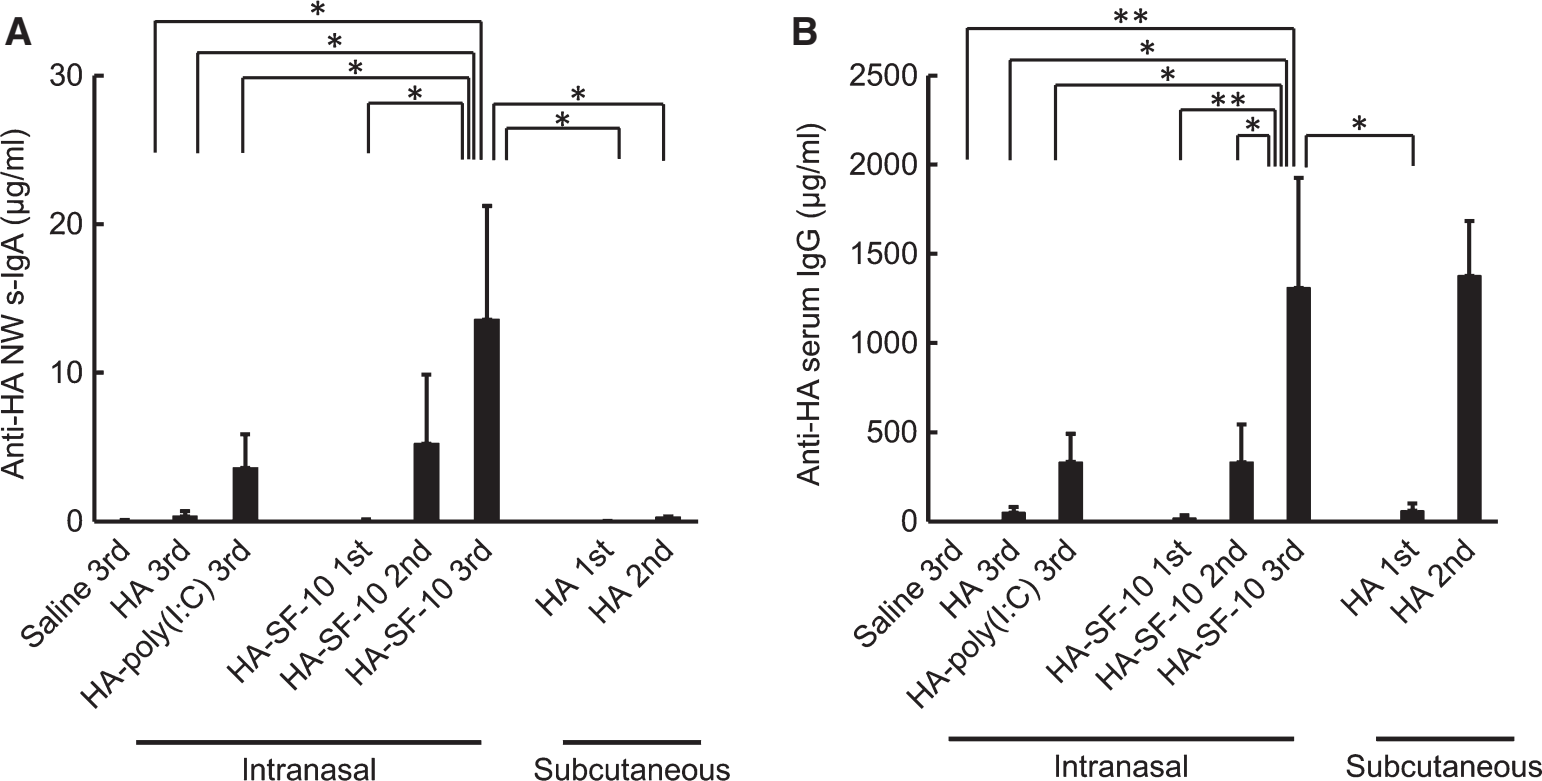
Comparison of the effects of intranasal immunization with HA–SF–10 on mucosal and systemic immunity with those of poly(I:C) and subcutaneous administration of HA. Mice received intranasal HA (0·2 μg) combined with or without SF‐10 (2 μg), poly(I:C) (2 μg), or saline, 2 or 3 times every 2 weeks. Another group of mice received subcutaneous HA once or twice every two weeks. After boost inoculation at 2 weeks, the levels of anti‐HA‐specific s‐IgA in nasal washes (NW) (A) and anti‐HA‐specific IgG in sera (B) were measured. Data are mean ± SD of 5–10 mice. *P < 0·05, **P < 0·01.

For the possible clinical use of SF‐10, we evaluated the adjuvanticity of HA–SF–10 and its protective immunity compared with subcutaneously administered HA and intranasally administered HA‐poly(I:C), which is one of the most potent mucosal vaccines reported,^20^ as positive controls (Figure 1). HA administered subcutaneously twice‐effectively induced anti‐HA‐specific IgG in sera, but not s‐IgA in nasal washes. In contrast, intranasally administered HA‐SF‐10 significantly induced both anti‐HA‐specific s‐IgA in nasal washes and anti‐HA‐specific IgG in sera, when immunization was repeated three times, compared with the levels induced by intranasal administration of HA or saline three times. No further increase in the induction was observed by administration of HA–SF–10 four times (data not shown). In addition, the levels of anti‐HA‐specific s‐IgA in nasal washes and anti‐HA‐specific IgG in sera induced by intranasally administered HA–SF–10 were significantly higher at 3·7‐ and 3·9‐fold, respectively, than those induced by intranasally administered HA‐poly(I:C) three times (*P *< 0·05). Anti‐HA‐specific IgG levels in sera induced by intranasal administration of HA–SF–10 three times were almost equivalent to those induced by twice subcutaneous administration of HA. The mean HI titers in serum induced by intranasal administration of HA–SF–10 three times were >300 and were in the similar ranges to those induced by subcutaneous administration of HA twice (Figure S1).

### Neutralization activities of nasal washes and protective immunity induced by HA–SF–10

**Figure 2:**
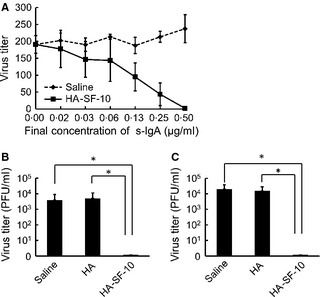
Neutralization activity and protective immunity of nasal washes in mice immunized with intranasal HA–SF–10. (A) Neutralization of IAV/New Caledonia/20/99(H1N1) by isolated s‐IgA fraction from nasal washes of mice immunized with intranasal HA–SF–10 or saline. Data are mean virus titer ± SD of three different experiments. (B and C) Two weeks after the last immunization, mice were infected with 5 × 10^4^ PFU of IAV/New Caledonia/20/99(H1N1), and virus titers in nasal washes (B) and lung washes (C) were measured at day 4 after infection. Data are mean ± SD of 8–10 mice. *P < 0·05.

In the next step, we assessed the neutralization activities of nasal washes. The neutralizing activity of nasal‐wash s‐IgA fraction after intranasal immunization with HA–SF–10 is shown in Figure 2A. Nasal‐wash s‐IgA of mice immunized intranasally with HA–SF–10 neutralized the inoculated IAV/New Caledonia/20/99(H1N1) in a dose‐dependent manner, but the fraction obtained from mice treated with saline did not show such activity.

**Figure 3:**
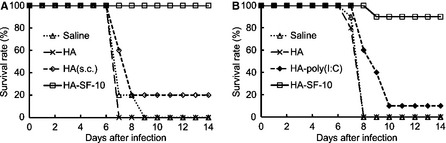
Survival rates of immunized mice after infection with lethal doses of IAV/PR8/34(H1N1) virus. (A) Mice were immunized with intranasal HA (0·2 μg) combined with or without SF‐10 (2 μg), polyc (I:C) (2 μg) or saline three times every 2 weeks. Another group of mice received subcutaneous (s.c.) HA twice every two weeks. Two weeks after the last immunization, mice were infected with 10 × LD_50_ (50 PFU) (A) and 160 × LD_50_ (800 PFU) (B) of IAV/PR8/34(H1N1) virus, and their survival rates were monitored.

We confirmed the protective immunity in virus challenge experiments (Figure 2B,C). The virus titers in nasal and lung washes were measured after 4‐day infection of immunized mice with 5 × 10^4^ PFU of IAV/New Caledonia/20/99(H1N1). High virus titers were detected in mice immunized with saline or intranasal HA. In contrast, the virus titer was below the detection limit in both nasal and lung washes of mice immunized intranasally with HA–SF–10. We also assessed the protective immunity in mice challenged with two lethal doses (10 × LD_50_, 50 PFU and 160 × LD_50_, 800 PFU) of IAV/PR8/34(H1N1), a similar subtype but highly pathogenic strain of IAV (Figure 3). All mice that were immunized intranasally with HA–SF–10 survived against the infection with 10 × LD_50_ of IAV/PR8/34(H1N1), but all mice that were immunized with intranasal HA or saline died at day 10. About 80% of mice immunized twice with subcutaneous HA died at day 8 (Figure 3A). Even in more severe virus challenge, 90% of mice immunized with intranasal HA–SF–10 survived against infection with 160 × LD_50_ of IAV/PR8/34(H1N1), while only 10% of mice immunized intranasally with HA‐poly(I:C) and none of those immunized intranasally with HA or saline survived (Figure 3B).

### Induction of HA‐specific Th1‐ and Th2‐type immune responses by HA–SF–10

**Figure 4:**
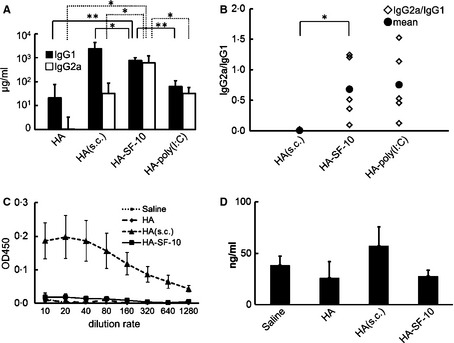
Induction of HA‐specific Th1‐ and Th2‐type immune responses by intranasal administration of HA–SF–10. Mice (*n* = 5) were immunized with intranasal HA (0·2 μg) combined with or without SF‐10 (2 μg) or poly(I:C) (2 μg) three times every 2 weeks. Another group of mice received subcutaneous (s.c.) HA (0·2 μg) twice every 2 weeks. Two weeks after the last immunization, sera were collected, and anti‐HA‐specific IgG1, IgG2a, IgE, and total IgE levels were measured by ELISA. (A) Data are mean ± SD of IgG1 and IgG2a in sera. *P < 0·05, **P < 0·01. (B) Anti‐HA‐specific IgG2a/IgG1 ratio in sera of individual mice and their mean values. *P < 0·05. (C) Induced anti‐HA‐specific IgE levels in each animal group. Data (OD 450) are mean ± SD of each serum dilution rate. (D) Data are mean ± SD of total IgE concentrations in each animal group.

To assess the effect of intranasal administration of HA–SF–10 on T‐helper responses, we analyzed the subclasses of anti‐HA‐specific IgG (IgG1 and IgG2a) as well as IgE in sera and the induction of Th1‐ and Th2‐type cytokine responses in mucosal lymphoid tissues. Intranasal immunization with HA–SF–10 induced not only anti‐HA‐specific IgG1 and Th2‐type antibody, but also anti‐HA‐specific IgG2a and Th1‐type antibody, and these levels suggested a balanced Th1/Th2 response (Figure 4A). The induced levels of IgG1 and IgG2a by intranasal administration of HA‐SF‐10 were significantly higher than those by HA‐poly (I:C) (*P *< 0·01 and *P *< 0·05, respectively). However, the mean serum IgG2a/IgG1 ratio in mice immunized intranasally with HA–SF–10 was comparable with that of mice immunized with HA‐poly(I:C) (Figure 4B). On the other hand, the mean serum IgG2a/IgG1 ratio was significantly low in mice immunized subcutaneously with HA compared with the value induced by HA–SF–10 (*P *< 0·05).

**Figure 5:**
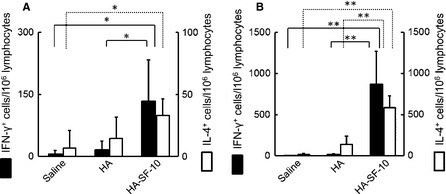
Induction of Th1‐ and Th2‐type cytokines in airway lymphocytes by intranasal administration of HA–SF–10. Mice (*n* = 5) were immunized with intranasal HA (0·2 μg) combined with or without SF‐10 (2 μg) or saline three times every 2 weeks. Two weeks after the last immunization, lymphocytes of NALT (A) and nasopharynx (B) were isolated and incubated with HA (5 μg/ml) for 16 hour. IFN‐γ‐ and IL‐4‐producing lymphocytes were measured by ELISPOT. Data are mean ± SD of cytokine‐producing lymphocytes per 10^6^ lymphocytes in 4–5 independent experiments. *P < 0·05, **P < 0·01.

Finally, we analyzed anti‐HA‐specific IgE (Figure 4C) and total IgE levels (Figure 4D) to exclude allergic responses after intranasal immunization with HA–SF–10. None of the mice immunized intranasally with HA–SF–10 showed the induction of anti‐HA‐specific IgE or total IgE in the serum, although distinct induction by subcutaneous administration of HA was detected. We also studied the expression levels of IFN‐γ (Th1‐associated cytokine) and IL‐4 (Th2‐associated cytokine) in mucosal lymphoid tissues of mice, such as NALT and nasopharynx, by ELISPOT (Figure 5). Mice immunized intranasally with HA–SF–10 showed significantly higher induction of both IL‐4‐ and IFN‐γ‐producing cells than the induction of mice immunized intranasally with HA or saline. The numbers of induced IL‐4‐ and IFN‐γ‐producing cells were higher in nasopharynx than those in NALT. The number of induced IL‐4‐producing cells and IFN‐γ‐producing cells supports the results of the balanced serum IgG2a/IgG1 ratio shown in Figure 4.

## Discussion

Many potential mucosal adjuvants have been evaluated in the past, but no safe and efficacious mucosal adjuvant is available commercially at present. To overcome these issues, we recently reported a bovine pulmonary surfactant that enhances antigen delivery to dendritic cells, increases antigen sustainability in the nasal cavity, and induces both mucosal and systemic immunity.^9^, ^14^, ^15^ To provide ample supply of this adjuvant without any risk of bovine spongiform encephalopathy, we prepared synthetic SSF, which comprises three major lipids, and synthetic human SP‐C(1–35), resembling the natural surfactant.^9^ Although the mucosal adjuvanticity of SSF is almost equivalent to that of natural Surfacten, there are four major problems that curtail the use of SSF clinically: (i) Although SP‐C(1–35) is an essential part of SSF, this hydrophobic peptide is insoluble in common organic solvents for large‐scale manufacturing. In the present study, we identified a peptide from the SP‐C‐related peptides with stable adjuvanticity and solubility in methanol as a substitute for human SP‐C(1–35). (ii) To further increase the antigen delivery efficacy of SSF, we increased HA binding to SSF at ≥92% by lyophilization. (iii) As HA and SSF mixing in a sonic oscillator is difficult to apply in industrial processing, we designed a processing method instead of sonication for large‐scale manufacturing. (iv) As mucosal adjuvanticity of natural Surfacten or SSF was less potent than that of poly(I:C) (Table 2 and Figure 1), we increased the efficacy of SSF adjuvanticity by further modification.

In the present study, we solved four major problems of SSF stated above and developed a potent synthetic adjuvant, SF‐10. Among the active SP‐C‐related peptides (Tables 1 and 2), we selected K6L16 as a substitute for human SP‐C(1–35), because K6L16 is soluble in methanol and expresses high adjuvanticity. To achieve more efficient interaction between HA and SSF and more efficient antigen delivery of SSF, they were mixed at 42°C, the critical temperature for surfactant lipids, for 10 minute, followed by lyophilization to remove water molecules between them, as a substitute for sonication. As shown in Table 3, lyophilization increased the binding of HA to SSF by ≥92% and markedly increased mucosal and systemic immunity. Lyophilization also seems to have two other benefits: protection against sonication‐related loss of heat‐labile HA antigenicity and large‐scale manufacturing.

We added CVP to HA‐SSF mixture at 0·5% to increase viscosity. The results showed the HA‐SSF mixture prepared by lyophilization had higher adjuvanticity than that prepared by sonication (Table 3). The addition of CVP to the HA‐SSF resulted in further increase in nasal‐wash s‐IgA production, probably due to the prolonged antigen presentation in the nasal cavity. Taken these improvements together, HA–SF–10 increased the induction of anti‐HA‐specific s‐IgA in nasal washes and anti‐HA‐specific IgG in sera compared with intranasal HA‐poly(I:C) and subcutaneous administration of HA (Figure 1). These data were supported by the neutralization activities in nasal washes, HI titers in serum, protective immunity, and high survival rates of animals immunized with intranasal HA–SF–10 (Figures 2 and 3, and Figure S1).

It has been reported that intranasal administration of the most potent toxin‐based mucosal adjuvant of cholera toxin (CT) or *Escherichia coli* heat‐labile enterotoxin (HLT) induces both nasal‐wash s‐IgA and serum IgG at about 3·8‐fold of those by poly (I:C).^21^ The data suggest that the efficacy of mucosal adjuvanticity of SF‐10 is similar to that of CT and HLT, because nasal‐wash s‐IgA and serum IgG induced by HA–SF–10 were about 4‐fold those induced by HA‐poly (I:C) (Figure 1). Importantly, application of virosomal influenza vaccine adjuvanted with HLT resulted in high incidence of Bell's palsy^8^ and CT‐induced IgE against antigen and CT.^22^ In contrast, SF‐10 did not result in such adverse reactions in animal experiments. Intranasal mucosal live attenuated virus vaccines, FluMist^®^ and FLUENZ, are currently available on the market. In a related issue, concern has been raised regarding the safety of FluMist^®^ in young children aged<2 years with previous asthma or with recurrent wheezing.^11^, ^12^ Although not tested yet, HA–SF–10 influenza vaccine could be potentially useful in young children, because Surfacten^®^ has been used in premature babies without significant adverse effects^13^ and is known to enhance systemic and mucosal immunity in minipigs even just after weaning.^15^

For the development of effective and safe mucosal vaccine, it is important that the mucosal adjuvant induces a balanced Th1‐ and Th2‐type cytokine response to support antigen‐specific antibody production (Figure 4) without inflammatory or allergic side effects.^23^, ^24^ Figure 5 shows that intranasal immunization with HA–SF–10 elicited anti‐HA‐specific Th1(IFN‐γ)‐ and Th2(IL‐4)‐type cytokine responses in the nasopharynx and NALT, compared with HA and saline. HA–SF–10 also induced a balanced Th1‐ and Th2‐type cytokine response in the airway mucosa. Of note, there was no detectable anti‐HA‐specific IgE and total IgE response in the sera of animals immunized intranasally with HA–SF–10. These results confirm that intranasal HA–SF–10 induces a balanced Th1‐ and Th2‐type cytokine response without the risk of allergy.

IAV‐specific CD8^+^ cytotoxic T lymphocytes and IFN‐γ‐producing CD4^+^ T lymphocytes promote clearance of IAV and recovery from infection.^5^ Intranasal HA–SF–10 activated IFN‐γ‐producing lymphocytes in the nasopharynx and NALT and probably stimulated cellular immunity against IAV. Considered together, our results indicate that intranasal immunization with HA–SF–10 provided efficient protection against IAV infection and markedly increased survival rates even in mice with fulminant viral infection. Administration of antigen‐SF‐10 by other mucosal routes should be evaluated in future studies.

## Conflict of interest

HK and DM are inventors of an applied patent related to the technology described in this study (Preparation methods of SF‐10 adjuvant; application number WO 2011/108521 A1), which is owned by the University of Tokushima. All remaining authors declare no conflict of interest.

## Supporting information


**Figure S1.** HI activity in sera of mice immunized with intranasal administration of HA–SF–10, HA and saline and with subcutaneous administration of HA.
