# Supplementary figures and images for: Intranasal influenza vaccination using a new synthetic mucosal adjuvant SF‐10: induction of potent local and systemic immunity with balanced Th1 and Th2 responses
Source: Influenza Other Respir Viruses. 2013 May 26;7(6):1218–26. doi: 10.1111/irv.12124 (PMC3933764; doi:10.1111/irv.12124)

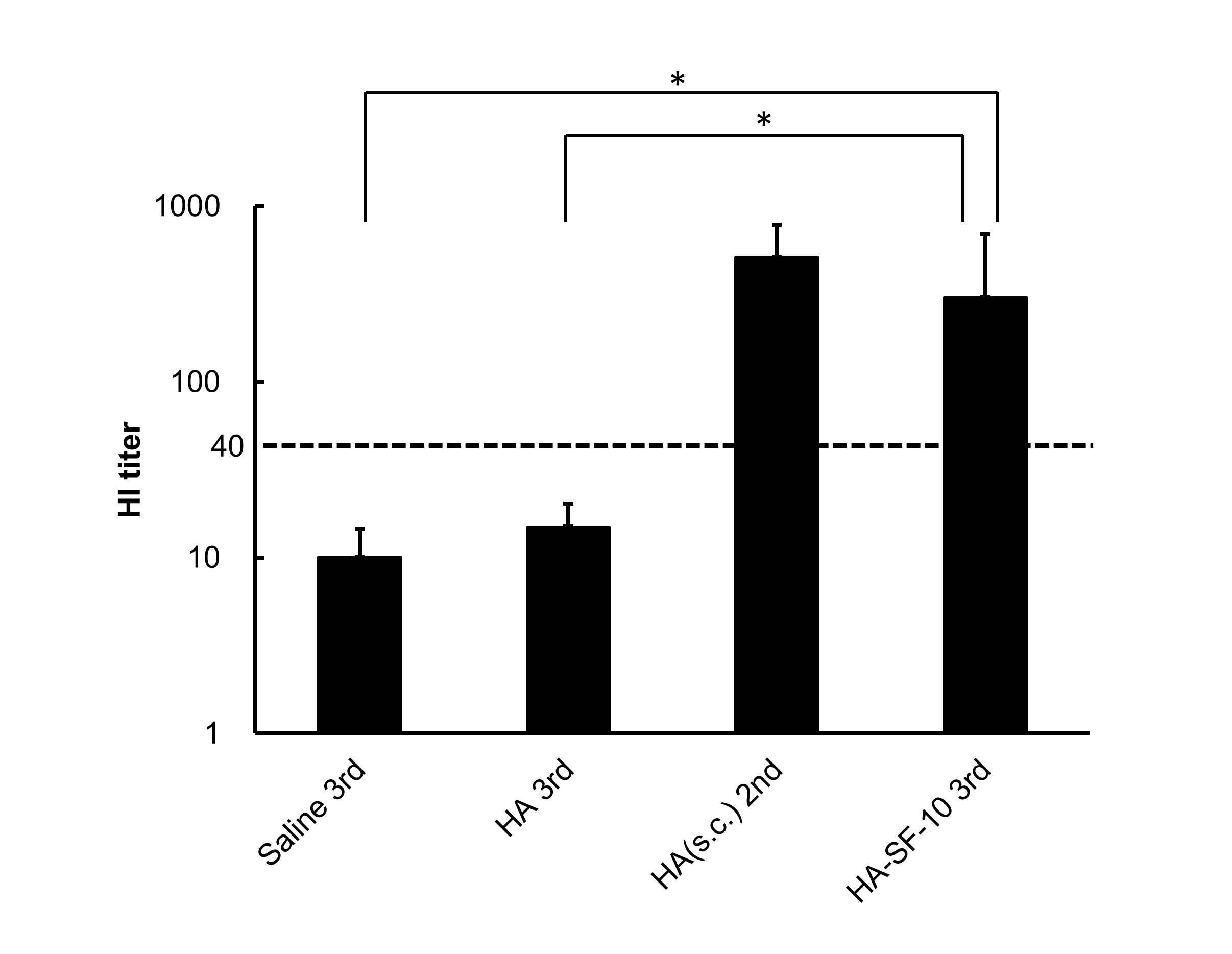

Supplement: Supplementary file 1 — Figure S1. HI activity in sera of mice immunized with intranasal administration of HA–SF–10, HA and saline and with subcutaneous administration of HA. [file IRV-7-1218-s001.TIF]
